# Accuracy of Heart Disease Prevalence Estimated from Claims Data Compared With an Electronic Health Record

**DOI:** 10.5888/pcd9.120009

**Published:** 2012-08-23

**Authors:** Thomas E. Kottke, Courtney Jordan Baechler, Emily D. Parker

**Affiliations:** Author Affiliations: Courtney Jordan Baechler, Division of Cardiology, Department of Medicine, University of Minnesota, Minneapolis, Minnesota; Emily D. Parker, HealthPartners Research Foundation, Minneapolis, Minnesota.

## Abstract

**Introduction:**

We developed a decision support tool that can guide the development of heart disease prevention programs to focus on the interventions that have the most potential to benefit populations. To use it, however, users need to know the prevalence of heart disease in the population that they wish to help. We sought to determine the accuracy with which the prevalence of heart disease can be estimated from health care claims data.

**Methods:**

We compared estimates of disease prevalence based on insurance claims to estimates derived from manual health records in a stratified random sample of 480 patients aged 30 years or older who were enrolled at any time from August 1, 2007, through July 31, 2008 (N = 474,089) in HealthPartners insurance and had a HealthPartners Medical Group electronic record. We compared randomly selected development and validation samples to a subsample that was also enrolled on August 1, 2005 (n = 272,348). We also compared the records of patients who had a gap in enrollment of more than 31 days with those who did not, and compared patients who had no visits, only 1 visit, or 2 or more visits more than 31 days apart for heart disease.

**Results:**

Agreement between claims data and manual review was best in both the development and the validation samples (Cohen’s κ, 0.92, 95% confidence interval [CI], 0.87–0.97; and Cohen’s κ, 0.94, 95% CI, 0.89–0.98, respectively) when patients with only 1 visit were considered to have heart disease.

**Conclusion:**

In this population, prevalence of heart disease can be estimated from claims data with acceptable accuracy.

## Introduction

We developed a spreadsheet–based decision support tool that helps the user determine which heart disease prevention and treatment interventions would be expected to have the biggest effect on mortality in a population (1). This tool can assist in nationwide efforts to control the prevalence of heart disease — for example, The Million Hearts initiative (2), *Healthy People 2020* (3), and the American Heart Association 2020 goals for disease control (4) and disease surveillance (5) — by identifying the interventions that are expected to have the greatest impact on deaths among populations. The decision support tool can also help direct regional and local initiatives.

Populating the decision support tool with US data, we found that implementing more effective primary and secondary prevention services could prevent or postpone as many as 56% of all deaths among people aged 30 to 84 years (1). In contrast, optimizing care for patients in this same age group who are hospitalized for coronary heart disease or heart failure would prevent or postpone approximately 8% of all deaths. The same finding is true for Lithuania, one of the Baltic countries: even with big opportunities to increase the intensity of care for acute events, interventions that prevent and control heart disease risk factors would more effectively reduce deaths (6).

The Institute of Medicine report on a nationwide framework for surveillance of cardiovascular and chronic lung diseases observed that, as electronic health records become more ubiquitous and health information exchanges become operational, they could become powerful tools for improving health and relieving the burden of chronic diseases (7). After extracting data from the electronic health records of a single medical group, we also used the decision support tool to identify opportunities to prevent or postpone deaths among patients being treated for heart disease (8). We found that the greatest opportunity to prevent or postpone deaths — 70% of the total opportunity — lies with optimizing care for ambulatory patients. Optimizing care from levels being delivered for patients hospitalized for acute myocardial infarction makes up only approximately 3% of the total opportunity to prevent or postpone deaths in the population.

To identify the magnitude of the opportunities among the population with chronic heart disease, disease prevalence must be known. Health plan claims data are a potential source of prevalence data, but disparities in length of enrollment and gaps in coverage are threats to the validity of prevalence calculations. To test the extent to which claims data are affected by these issues, we compared diagnosis based on claims data to diagnosis based on manual record review in a stratified random sample of a population enrolled in a health plan and treated by the associated medical group.

## Methods

This record review was approved by the HealthPartners Research Foundation institutional review board on November 20, 2008, as protocol number 08–093. The study was completed on December 29, 2011, and was conducted in Minneapolis, Minnesota.

### Selection of the sample

The study sample was drawn from people aged 30 years or older who were covered by HealthPartners insurance and received any type of care from HealthPartners medical group. We characterized these people by 3 attributes: any length of enrollment versus long enrollment, a gap in enrollment, and number of visits for heart disease. The “any enrollment” group comprised patients who were enrolled at any time from August 1, 2007, through July 31, 2008, and may have been enrolled on August 1, 2005. The “long enrollment” group comprised patients who were enrolled at any time from August 1, 2007, through July 31, 2008, and were also enrolled on August 1, 2005. Patients were considered to have a gap in enrollment if they had an enrollment gap of more than 31 days during August 1, 2007, through July 31, 2008. Three patterns of visits for heart disease (*International Classification of Diseases, Ninth Revision, Clinical Modification* [ICD-9-CM] codes 410–414, 420–429, or both) were identified: no visits, at least 1 visit for heart disease without 2 or more visits separated by an interval greater than 31 days, or 2 or more visits more than 31 days apart for heart disease. From this population we selected a stratified random sample of 480 patients and randomly allocated them to 2 subsamples. One subsample served as the development sample for the analysis and the other subsample served as the validation sample.

### Record review protocol

A cardiologist (T.E.K. or C.J.B.) manually reviewed the record of each patient for evidence that the patient had heart disease. To increase the probability of detecting references to heart disease in the free-text portion of the record, we used the electronic health record text search function to search all records for the following words: angiogram, atherosclerosis, bypass, cardiac stress test, coronary, echocardiogram, ejection fraction, heart attack, heart disease, heart failure, infarct, ischemic heart disease, myocardial perfusion, and sestamibi. We also searched the records for the following acronyms: AMI (acute myocardial infarction), CABG (coronary artery bypass graft), CAD (coronary artery disease), CHD (coronary heart disease), CHF (congestive heart failure), CVD (cardiovascular disease), and MI (myocardial infarction). We accepted the following as evidence of heart disease: tests that were positive for heart disease, a clinic visit during which heart disease was treated, heart disease mentioned in the past medical history, a hospital discharge coded for heart disease, or heart disease on a problem list. We did not require objective evidence of heart disease. However, if the sole evidence for heart disease was a heart disease code for a test that was performed while the patient was hospitalized (eg, an echocardiogram), the code was not accepted as evidence that the patient had heart disease. For the cases in which the manual review disagreed with claims data, we manually reviewed the record a second time to determine whether a reference to heart disease in the text had been overlooked. To test the reproducibility of the manual record review, both cardiologists abstracted a stratified random sample of 48 records. They agreed on the classification of 47 of the 48 records.

### Statistical analysis

We calculated Cohen’s κ and the 95% confidence interval (CI) for patients who had been enrolled any time from August 1, 2007, through July 31, 2008. We performed the calculations for both the development and validation samples. Because it has been suggested that accuracy requires 2 visits for heart disease (9,10), we calculated κ values for 2 classification scenarios: a scenario in which patients with only 1 visit would be classified as not having heart disease and an alternative scenario in which these same patients were classified as having heart disease. We also calculated sensitivity and specificity for these 2 scenarios and calculated positive and negative predictive values for the reconstructed population.

We used logistic regression to test whether any of the variables used in the analysis were associated with agreement between claims data and manual review. Agreement was the dependent variable and age, sex, duration of enrollment, gap in enrollment, and number of visits for heart disease were the independent variables. The development data set and the validation data set were combined to perform a single data set for the analysis. Because agreement between claims data and manual record review for patients with no visits for heart disease was perfect, we excluded these patients from the multivariate analysis.

## Results

### Demographic characteristics of the population

Just over half of the 474,089 people in the population were women, and the average age was slightly less than 50 years (Table 1). The long enrollment subpopulation consisted of a higher proportion of women and was slightly older on average than the any enrollment population. Compared with people who had a gap in enrollment, people without a gap in enrollment were more likely to be female and older. People in the subpopulation without any visits for heart disease were more likely to be female and younger than people who had 1 or more visits for heart disease. People who had multiple visits for heart disease tended to be older and were more likely to be female than people who had only 1 visit for heart disease. Prevalence of heart disease in the population with any length of enrollment was 7.9%; prevalence of heart disease in the subpopulation that was also enrolled on August 1, 2005, was 10.3%.

**Table 1:** Demographic Characteristics of the Study Population Enrolled in an Insurance Plan by Duration of Enrollment, Gaps in Enrollment, and Number of Visits Coded for Heart Disease, Minneapolis, Minnesota, 2007–2008^a^

Length of Enrollment/Gap in Enrollment >31 Days	No. of Visits for Heart Disease^b^ Based on Claims Data
**Enrolled any time from August 1, 2007–July 31, 2008; N = 474,089; female, 52.4%; age, 49.4 (18.0)**	

Gap: n = 146,939; female, 50.2%; age, 46.1 (16.0)	None: n = 140,440; female, 53.9%; age, 44.5 (15.0)
	1 visit: n = 3,918; female, 38.6%; age, 60.6 (21.0)
	≥2 visits: n = 2,581; female, 40.5%; age, 64.6 (23.0)

No gap: n = 327,150; female, 53.4%; age, 51.0 (17.0)	None: n = 296,073; female, 56.3%; age, 45.8 (17.0)
	1 visit: n = 15,182; female, 47.1%; age, 62.5 (22.0)
	≥2 visits: n = 15,895; female, 50.6%; age, 68.3 (20.0)

**Also enrolled on August 1, 2005; N = 272,348; female, 54.0%; age, 52.0 (18.0)**	

Gap: n = 40,174; female, 52.6%; age, 48.7 (18.0)	None; n = 37,179; female, 57.7%; age, 48.7 (18.0)
	1 visit: n = 1,601; female, 45.4%; age, 71.6 (24.0)
	≥2 visits: n = 1,394; female, 47.0%; age, 75.7 (22.0)

No gap: n = 232,174; female, 54.2%; age, 52.6 (17.0)	None: n = 207,195; female, 58.1%; age, 53.1 (17.0)
	1 visit: n = 11,782; female, 43.7%; age, 69.1 (21.0)
	≥2 visits: n = 13,197; female, 43.2%; age, 72.3 (19.0)

^a^ Ages are presented in years and as mean (interquartile range).

^b^ *International Classification of Diseases, Ninth Revision, Clinical Modification* codes 410–414, 420–429, or both.

### Agreement in the development sample

Classification based on claims data agreed with the manual review classification for all but 9 of the 240 records examined in the development sample (Table 2). All 9 disagreements were false positives and occurred in the records of patients who were not in the long enrollment group and had only 1 visit for heart disease. Seven of the disagreements were among patients with no gap in enrollment, and 2 of the disagreements were among patients with a gap in enrollment. If the prevalence heart disease is recalculated on the basis of the manual record review, it is 6.7% rather than 7.3% in the any enrollment population. Prevalence remained 10.3% in the long enrollment population.

**Table 2:** Agreement and Disagreement Between Claims Data and Manual Record Review for the Development Set^a^, Minneapolis, Minnesota, 2007–2008

Enrollment Category	Enrollment Gap >31 days	No. of Visits for Heart Disease Based on Claims Data	Agreement in the Development Sample (95% CI)	Agreement in the Validation Sample (95% CI)
Enrolled any time from August 1, 2007–July 31, 2008	Gap	0	1.00 (0.90–1.00)	1.00 (0.90–1.00)
		1	0.90 (0.69–0.98)	0.90 (0.69–0.98)
		≥2	1.00 (0.90–1.00)	0.95 (0.76–0.99)

	No gap	0	1.00 (0.90–1.00)	1.00 (0.90–1.00)
		1	0.65 (0.43–0.82)	0.85 (0.63–0.96)
		≥2	1.00 (0.90–1.00)	0.85 (0.63–0.96)

Also enrolled on August 1, 2005	Gap	0	1.00 (0.90–1.00)	1.00 (0.90–1.00)
		1	1.00 (0.90–1.00)	0.90 (0.69–0.98)
		≥2	1.00 (0.90–1.00)	0.95 (0.76–0.99)

	No gap	0	1.00 (0.90–1.00)	1.00 (0.90–1.00)
		1	1.00 (0.90–1.00)	1.00 (0.90–1.00)
		≥2	1.00 (0.90–1.00)	1.00 (0.90–1.00)

Abbreviation: CI, confidence interval.

^a^ Twenty records were audited for each of the 12 categories for number of visits.

Cohen’s κ was 0.74 (95 % CI, 0.66–0.82) when patients with only 1 visit were considered not to have heart disease. The true number of cases of heart disease was underestimated by 13,395. Estimated prevalence of heart disease was 3.9% with this assumption, an underestimation of 42%. When record review was the gold standard, the sensitivity of ICD coding was 0.53 (95% CI, 0.45–0.61) and specificity was 1.00 (95% CI, 0.95–1.00). The predictive value of a positive test (PV+) was 1.0 and the predictive value of a negative test (PV−) was 0.08.

Cohen’s κ was 0.92 (95% CI, 0.87–0.97) when patients with only 1 visit for heart disease were classified as having heart disease. The true number of cases was overestimated by 5,706. Estimated prevalence of heart disease was 7.9% with this assumption, an overestimation of 18%. Because there were no disagreements between the claims data and manual record review in the long enrollment sample, the estimate of heart disease prevalence (10.3%) was the same with both assumptions. When chart review was considered the gold standard, sensitivity of the ICD codes was 1.00 (95% CI, 0.98–1.00) and specificity was 0.94 (95% CI, 0.97–1.00). PV+ was 0.59, and PV− was 1.0.

### Agreement in the validation sample

Classification based on claims data agreed with classification based on manual review in all but 12 of the 240 records in the validation sample (Table 2). Seven of the disagreements were for patients who had only 1 visit for heart disease and 5 of the disagreements were for patients who had 2 or more visits more than 31 days apart. The disagreements were equally divided among patients with and without a gap in enrollment. Only 3 of the disagreements were among the long enrollment group of patients. If the proportion of patients with heart disease is recalculated on the basis of the manual record review, the prevalence of heart disease was 6.8% in the any enrollment population and 10.2% in the long enrollment population.

If patients who had only 1 visit were considered not to have heart disease, Cohen’s κ was 0.65 (95% CI, 0.56–0.74). The prevalence of heart disease was 3.9% in the any enrollment population, an underestimation of 43%, and the prevalence of heart disease in the long enrollment population was 5.4%, an underestimation of 48%. When chart review was considered the gold standard, sensitivity of the ICD codes was 0.51 (95% CI, 0.42–0.59), specificity was 0.95 (95% CI, 0.87–0.98), PV+ was 0.29, and PV− was 0.07.

Conversely, if patients with only 1 visit for heart disease were classified as having heart disease, Cohen’s κ was 0.94 (95% CI, 0.89–0.98) and the estimated prevalence of heart disease in the any enrollment population was 7.9%, an overestimation of 16%. Estimated prevalence of heart disease in the long enrollment population was 10.3%, an overestimation of 1%. When chart review was considered the gold standard, sensitivity of the ICD codes was 1.00 (95% CI, 0.98–1.00), specificity was 0.87 (95% CI, 0.78–0.93), PV+ was 0.40, and PV− was 1.0.

### Patient attributes associated with correct classification

When patients who had no visits for heart disease were excluded from a logistic regression analysis, long enrollment (*P* < .01) and 2 or more visits (*P* < .02) were significantly associated with agreement between the classification based on claims data and the classification based on manual record review. Presence or absence of a gap in enrollment between August 1, 2007, and July 31, 2008 (*P* = .35), sex (*P* = .07), and age (*P* = .86) were not.

## Discussion

This study investigated the effect of length of enrollment, gaps in coverage, and number of visits for heart disease on the agreement of claims data with manual record review. Agreement was higher for patients who had longer enrollment and more than 1 visit for heart disease, but even so, accepting claims data at face value yielded the highest levels of agreement and the best estimates of true prevalence. Requiring at least 2 outpatient visits to classify a patient as having heart disease resulted in heart disease prevalence being underestimated by nearly 50%, an error that significantly underestimates the potential effect of secondary prevention initiatives.

Our conclusion that claims data acceptably represent the presence or absence of disease is generally consistent with other analyses of administrative data (11–13). Lix et al, in Manitoba, concluded that Cohen’s κ associated either with a single physician visit in a year or a single hospitalization in a year was approximately equal (14). However, the κ values that Lix et al found were considerably less, 0.45 to 0.55, than those we found. Assessing the ability to identify patients with diabetes, investigators from the Indian Health Service concluded that their database can identify with acceptable accuracy the patients who have diabetes (12). A review of methods to identify patients with diabetes found that studies using either administrative data or survey data were both adequately sensitive and highly specific (13). Others have urged caution when using self-report of disease incidence or prevalence (15). We are not confident that claims data reflect disease incidence.

These conclusions must be accepted with some caveats. Perhaps foremost, the analysis is based on the records of only 1 medical group; other groups may have a different experience. Also, the purpose of this study was to determine the algorithm that most accurately estimates the prevalence of heart disease in an enrolled population. If the purpose were to identify patients for case management or to assess the quality of health care (9,10), requiring at least 2 outpatient visits or a hospitalization to conclude that a patient has heart disease is more prudent.

We also do not have an adequate explanation for the fact that heart disease prevalence was 50% higher in the long enrollment population than in the any enrollment population. It may be that patients who have heart disease are less likely to change health plans because of coverage limitations for pre-existing conditions or that they have a preference for a long-term relationship with a particular physician. The fact that the prevalence rates were nearly identical in the development and validation samples makes the observation less likely to be a data sampling error.

We acknowledge that the disease prevalence estimates based on claims data appear to overestimate true prevalence by 15% to 20%. However, assuming that the true prevalence of heart disease is 20% lower than the calculated prevalence does not substantively change the conclusion that improving secondary prevention (33% of the total opportunity) would have a far greater effect on deaths in the United States than would improving care for patients hospitalized for acute cardiac events (8% of the total opportunity) (1). Likewise, it does not change the conclusion that most of the opportunity to ease the burden of death for HealthPartners Medical Group patients with heart disease lies with improvement in ambulatory care (70% of the total opportunity) rather than improvement in care for acute myocardial infarction (3% of the total opportunity) (8).

It is probably safe to assume that coding practices vary between medical groups and that any medical group should document that their billing codes acceptably reflect the medical record before they use them to estimate disease prevalence. However, the data presented here suggest that claims data can be used to estimate disease prevalence.
